# Systemically transplanted mesenchymal stem cells induce vascular-like structure formation in a rat model of vaginal injury

**DOI:** 10.1371/journal.pone.0218081

**Published:** 2019-06-13

**Authors:** Ofra Ben Menachem- Zidon, Michal Gropp, Etti Ben Shushan, Benjamin Reubinoff, David Shveiky

**Affiliations:** 1 The Hadassah Human Embryonic Stem Cell Research Center, Goldyne Savad Institute of Gene Therapy, Hadassah - Hebrew University Hospital, Jerusalem, Israel; 2 Department of Obstetrics and Gynecology, Hadassah - Hebrew University Medical Center, Jerusalem, Israel; Università degli Studi della Campania, ITALY

## Abstract

The beneficial effect of mesenchymal stem cells (MSCs) on wound healing is mostly attributed to a trophic effect that promotes angiogenesis. Whether MSCs can contribute to the formation of new blood vessels by direct differentiation is still controversial. Pelvic floor dysfunction (PFD) is a group of disorders that negatively affect the quality of women’s lives. Traditional vaginal surgical repair provides disappointing anatomical outcome. Stem cell transplantation may be used to supplement surgery and improve its outcome. Here we aimed to examine the engraftment, survival, differentiation and angiogenic effect of transplanted MSCs in a vaginal injury rat model. MSCs were obtained from the bone marrow of Sprague Drawley (SD) rats, expanded and characterized *in vitro*. The MSCs expressed CD90 and CD29, did not express CD45, CD34, CD11b and CD31 and could differentiate into osteogenic, chondrogenic and adipogenic lineages. Cells were labeled with either PKH-26 or GFP and transplanted systemically or locally to female SD rats, just after a standardized vaginal incision was made. Engraftment after local transplantation was less efficient at all-time points compared to systemic administration. In the systemically transplanted animal group, MSCs migrated to the injury site and were present in the healed vagina for at least 30 days. Both systemic and local MSCs transplantation promoted host angiogenesis. Systemically transplanted MSCs created new vascular-like structures by direct differentiation into endothelium. These findings pave the way to further studies of the potential role of MSCs transplantation in improving surgical outcome in women with PFD.

## Introduction

Mesenchymal stem cells (MSCs) have been reported to possess a potential therapeutic effect in various human pathological conditions [1–3]. They can be isolated from several sources, including bone marrow [4], adipose tissue [5], muscles [6], dental pulp [7,8], endometrium [9] and umbilical cord [10,11].

Proper healing of a damaged tissue relies on the creation of new blood vessels [12]. The beneficial effect of MSCs on wound healing is mostly attributed to a trophic effect, by the secretion of growth factors that promote angiogenesis [7,13,14]. Recent studies explore the most suitable donor tissue for the isolation of MSCs as well as the optimal culture conditions for propagation of MSCs in order to obtain the best effect on angiogenesis post transplantation [7,11,15]. Whether MSCs can contribute to the formation of new blood vessels by direct differentiation is still controversial [11,16–20].

The neural crest is suggested as a potential origin of MSCs. Recent studies have shown that bone marrow MSCs exhibit phenotypes of neural crest cells [21,22]. Additional studies have shown the existence of neural crest cells in the adult dental follicle [23]. Upon differentiation, these cells efficiently gave rise to neurons, osteoblasts, adipocytes and endothelial cells [20,21,23]. These findings may explain the wide differentiation potential of MSCs and support the observations claiming endothelial differentiation of MSCs.

Pelvic floor dysfunction (PFD) is a group of common disorders that adversely affect women’s quality of life. These include pelvic organ prolapse (POP), stress urinary incontinence (SUI) and sexual dysfunction [24,25]. POP is an extremely prevalent condition, with over 300,000 surgical procedures for prolapse repair performed annually in the United States [26]. The majority of surgeries for POP repair are performed on older post-menopausal women, and reoperation rate was reported to be as high as 30%. Since traditional surgical methods yield suboptimal anatomical outcomes, there is a great clinical need to explore alternative treatment approaches.

Stem cell transplantation may be used as a biological therapeutic method supplementing the surgical treatments. MSCs transplantation has been studied in other pelvic floor conditions, such as SUI. MSCs were transplanted to the sub-urethral tissue in different animal models of PFD [27–29], including models of vaginal distention [30,31]. Nevertheless, previous studies on stem cell transplantation into the vaginal tissue displayed ambiguous results, with poor survival of the transplanted cells over time [32–38]. PFD is unique to humans and there is no authentic animal model for this condition. To address this major translational deficit we established a rodent model of vaginal injury that allows the study of vaginal wound healing processes.

In this study, we examined the engraftment, survival and differentiation of transplanted MSCs in the vaginal injury rat model.

## Materials and methods

This study was approved by the Hebrew University Animal Care and Use Committee. *Sprague Dawley* (SD) female rats were held at the SPF unit in Hadassah medical school with food and water *ad libitum*. All rats were 10 weeks old, with an average weight of 200gr, when operated.

### Isolation of bone marrow derived MSCs

MSCs were isolated from the tibia and femur bones of 10 weeks old female SD rats. Bone marrow was flushed with DMEM. Cells were resuspended and went through a 70μm cell strainer (BD Falcon) to remove bone debris and blood aggregates. Cells were then centrifuged at 4° Celsius, 1200 rpm for 5 minutes. The supernatant was removed and cells were resuspended with DMEM containing 10% FBS, 1% pen- strep and 1% glutamine (MSC medium, all products from Gibco). Ten (10) ml cell suspension was seeded into 10 cm culture petri dish for a total of two dishes in a 5% CO_2_, 37° Celsius incubator [39]. Medium was changed 24 hours afterwards and from then on, every 2–3 days.

### Characterization of MSCs by FACS analysis

Dissociation of the cells was done by trypsin- EDTA (Gibco). 100,000 cells were taken for the detection of each marker. Cells were briefly centrifuged and resuspended in 45 μl cold FACS buffer. The following conjugated antibodies were added for 30 minutes on ice: CD90.1-FITC and the isotype control: Mouse IgG2a- FITC, CD-29 with the hamster IgGλ FITC isotype, CD-45-PE, CD11b/c- PE and CD34- PE, CD31- PE, all four with the isotype control REA (S)-PE, (all antibodies from Miltenyi Biotec). Cells were then washed twice with FACS buffer and analyzed using FACS Calibur system (Becton Dickinson).

### Characterization of MSCs by multi- lineage differentiation

Cells were grown until passage 5–6 in MSCs medium. The cells were then transferred to specific medium conditions to induce differentiation into adipogenic, osteogenic and chondrogenic lineages. Evaluation of adipogenesis was done by Oil red O staining. For osteogenic differentiation, 6*10^4^ cells/well were seeded in 24- well plate. Cells were incubated for 21 days, with medium replacement every 3 days. Evaluation of osteogenesis was done with 2% Alizarin red S (Sigma). For chondrogenic differentiation, 1x10^5^ cells/well were seeded in 96-well U bottom culture plate. Spheroids were spontaneously formed within 24–48 hours and the medium was changed to MSCgo^™^ chondrogenic XF medium. Evaluation of chondrogensis was done with Alcian blue.

### Labeling the cells before transplantation

Cells were labeled with either PKH-26 or with green florescent protein (GFP). For PKH-26 labeling, cells were dissociated by trypsin EDTA and then centrifuged, washed and incubated with PKH-26 [40] according to the recommended protocol by Sigma (PKH26GL, Sigma). Positive labeling was verified using fluorescence microscopy just before transplantation. For GFP labeling, viruses were produced by transient co-transfection of three plasmids into 293T cells as described earlier [41,42], with several modifications. Briefly, 2 × 10^6^ 293T cells were transfected using the TransIT-293 Transfection Reagent (Mirus) with a total of 20μg of plasmid DNA: 3.5μg of the envelope plasmid pMD.G harboring the gene encoding VSV-G, 6.5μg of the packaging plasmid pCMVΔR8.91, and 10μg of the transfer vector expressing eGFP. The medium was replaced 24 hours after transfection with the MSC medium. The medium, which contains the viral particles, was collected 48 and 72 hours after transfection and filtered through 0.45-μm filters (Sartorius, Goettingen, Germany). The efficacy of the transfection was evaluated by FACS analysis.

### Animal model and procedure

Rats were anesthetized by a mixture of Ketamine and Dormitor (75 mg/kg BW and 0.5 mg/kg BW). A standardized posterior midline vaginal incision was performed. Rats were given Atipamezole (1mg/kg) at the end of the operation to reverse the anesthetic effects. MSCs were transplanted either systemically- intravenously (I.V.) to the tail vein, or locally, to the vaginal sub-epithelial fibro-muscular tissue at the injury site. Both groups were transplanted with 2*10^6^ cells.

The following groups were included in this study (n = five rats/ group):

1. I.V. MSC, which were transplanted with MSCs systemically, following vaginal incision.
2. Local MSC, which were transplanted with MSCs into the vaginal sub-epithelial fibro-muscular tissue, following vaginal incision.
3. Sham rats, which received medium only, either systemically or locally following vaginal incision.
4. No incision group, which were transplanted with MSCs systemically but no incision was made.
5. Naïve rats, which had no surgery and received no treatment.

Rats were monitored on a daily basis after the incision. According to the protocol, in case of excessive bleeding, rats were excluded from the experiment. In each treatment group, rats were sacrificed at three days, seven days and thirty days post transplantation (n = 5 rats/ group/ at each time point).

### Dissection of vaginal tissue and immunofluorescence evaluation

Rats were sacrificed with an overdose of CO2. The vagina was dissected and fixed with 4% PFA for one week. 6μm cuts were prepared and were taken for staining. The following primary antibodies were used: anti- GFP antibody ab6673 (1:100, abcam), anti- CD31 sc-376764 (1:100, Santa Cruz), anti laminin rb0821a (Thermi scientific, 1:100), mouse anti human muscle actin (1:100, Dako cytomation), anti -von Willebrand factor (vWF) bs-10048R (1:50, Bioss antibodies) and anti trimethyl-Histone H3 (Lys27) (1:200, Merck). Incubation with primary antibodies was for 24 hours in 4° Celsius. It was followed by three washes with 1xPBS (BI, Israel) and the appropriate secondary antibodies were added: donkey anti goat- 488, donkey anti mouse-594, donkey anti rabbit- 488 and donkey anti rabbit- 594 (all from Jackson laboratories).

### Quantification of blood vessels

Identification of blood vessels was done by immunostaining with the typical markers aSMA and CD31. In each group, one slide from the anterior, middle and posterior parts of the vagina, were taken for quantification (three slides were taken from each rat). In each slice, five different fields were arbitrary chosen for analysis. The average number of blood vessels in each field was calculated.

### Statistical analysis

All data (means ± standard errors of the means) were analyzed by multivariate analysis of variance, followed by post hoc tests. *P* values of <0.05 were considered significant.

## Results

### Characterization of MSCs *in vitro*

MSCs were characterized by their well-spread typical morphology after attachment to the culture dish (S1A–S1D Fig), by their capability to differentiate into multiple mesenchymal lineages and by the expression of markers.

MSCs could differentiate into the osteogenic (S1E Fig), chondrogenic (S1F Fig) and adipogenic lineages (S1C Fig) in three independent experiments (in triplicates, n = 9). The expression of surface markers of MSCs was assessed by FACS analysis, which was performed on cultures at passage 6–7 from five different experiments. It showed that 85±3.1% of the cells expressed CD90 and 88±2.2% expressed CD29 while there was negligible expression of CD34 (0.56±0.18%), CD45 (0.85±0.22%) and CD11b/c (0.8±0.19%) (S1I Fig). This pattern of marker expression is consistent with the suggested phenotype of MSCs [43,44].

### Healing of vaginal incision at different time points

**Fig 1:**
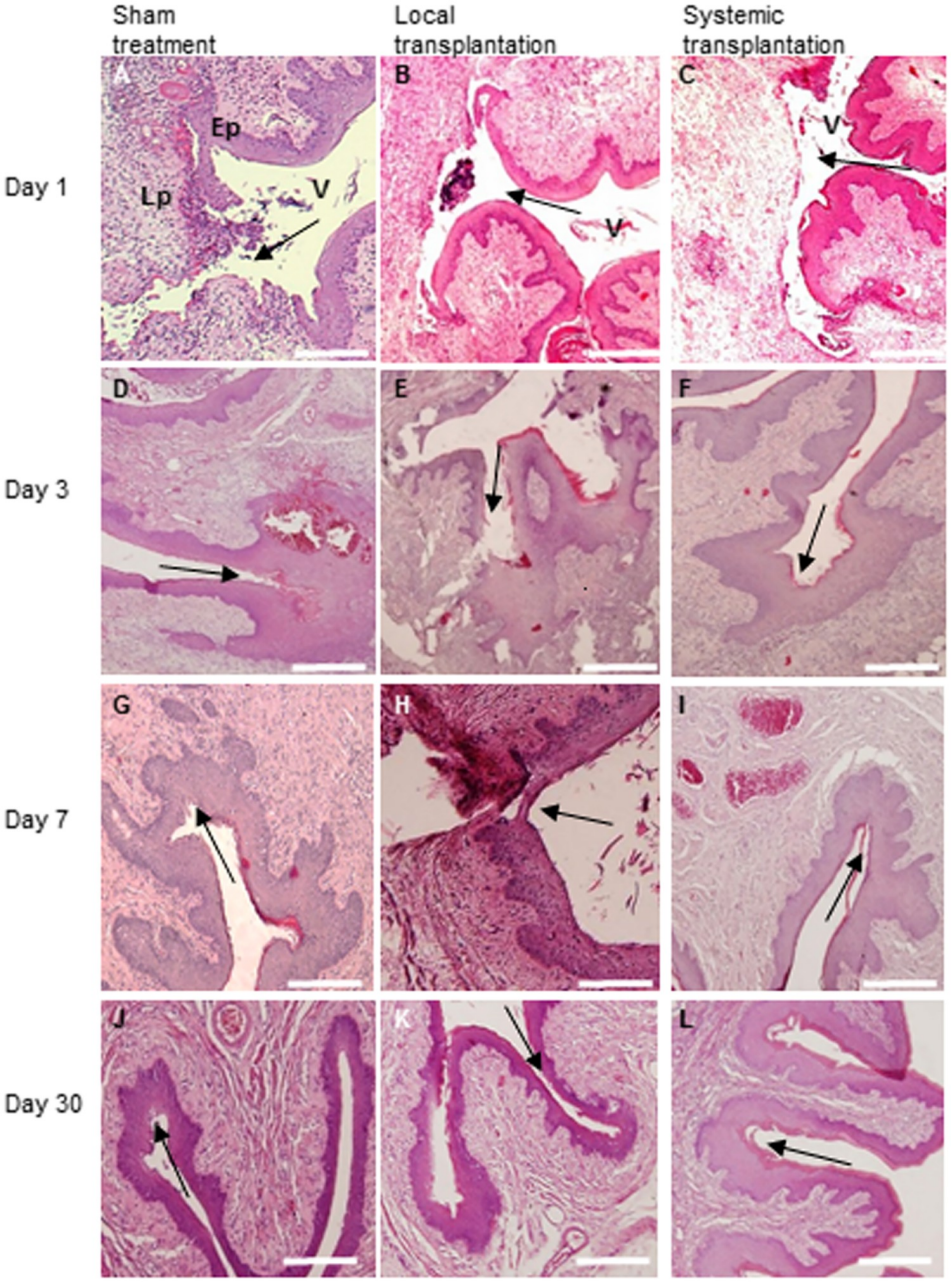
Healing of vaginal incision in sham, local and systemically transplanted rats. On day one post injury, disruption of the epithelial layer and the adjacent lamina propria was noted in all groups (A-C). On day three post injury, the epithelial cut was bridged but the layer was still thick in the sham (D) and I.V. (F) groups while it was disrupted in the local group (E). On day seven post injury, in the sham (G) and I.V. (I) treated rats the epithelial layer had a normal appearance while there was still partial epithelial disruption and disorganization of the sub-epithelial fibromuscular tissue in local transplanted rats (H). Complete re-epithelization was observed on day 30 in the sham (J), local (K), and I.V. (L) treated rats. n = 5 rats in each treatment group at each time point. The location of the incision is marked with the black arrow. Abbreviation: V = vagina; Ep = epithelium; Lp = lamina propria; Scale bar = 200μm.

A standardized vaginal incision was made in female SD rats. Cells were labeled either with PKH-26 or by infection with lentiviral vector constitutively expressing GFP. Both methods were highly efficient, as detected by fluorescence microscopy before transplantation. FACS analysis confirmed that 99% of infected cells expressed GFP (data not shown). Labelled MSCs were transplanted immediately after the injury either I.V. to the tail vein or locally, to the vaginal sub-epithelial fibro-muscular layer, at the injury site. Healing was analyzed and compared at sequential time points between the locally, I.V. transplanted and sham operated groups. Vaginal tissue was dissected at different time points post injury (n = five rats/ each time point) and the incision region, which was located opposite to the urethra, was analyzed in H&E stained cross sections. On day one post injury, disruption of the epithelial layer and the adjacent lamina propria was noted in all groups (Fig 1A–1C). On day three post injury, the epithelial cut was bridged, but the epithelial layer was still thick in the sham (Fig 1D) and I.V. (Fig 1F) groups while it was disrupted in the local group (Fig 1E). On day seven post injury, in the sham (Fig 1G) and I.V. (Fig 1I) treated rats the epithelial layer had a normal appearance while there was still partial epithelial disruption and disorganization of the sub-epithelial fibromuscular tissue in local transplanted rats (Fig 1H). On day 30, complete healing of the epithelial layer was observed in all groups (Fig 1J–1L).

### Survival of MSCs following local or systemic transplantation

**Fig 2:**
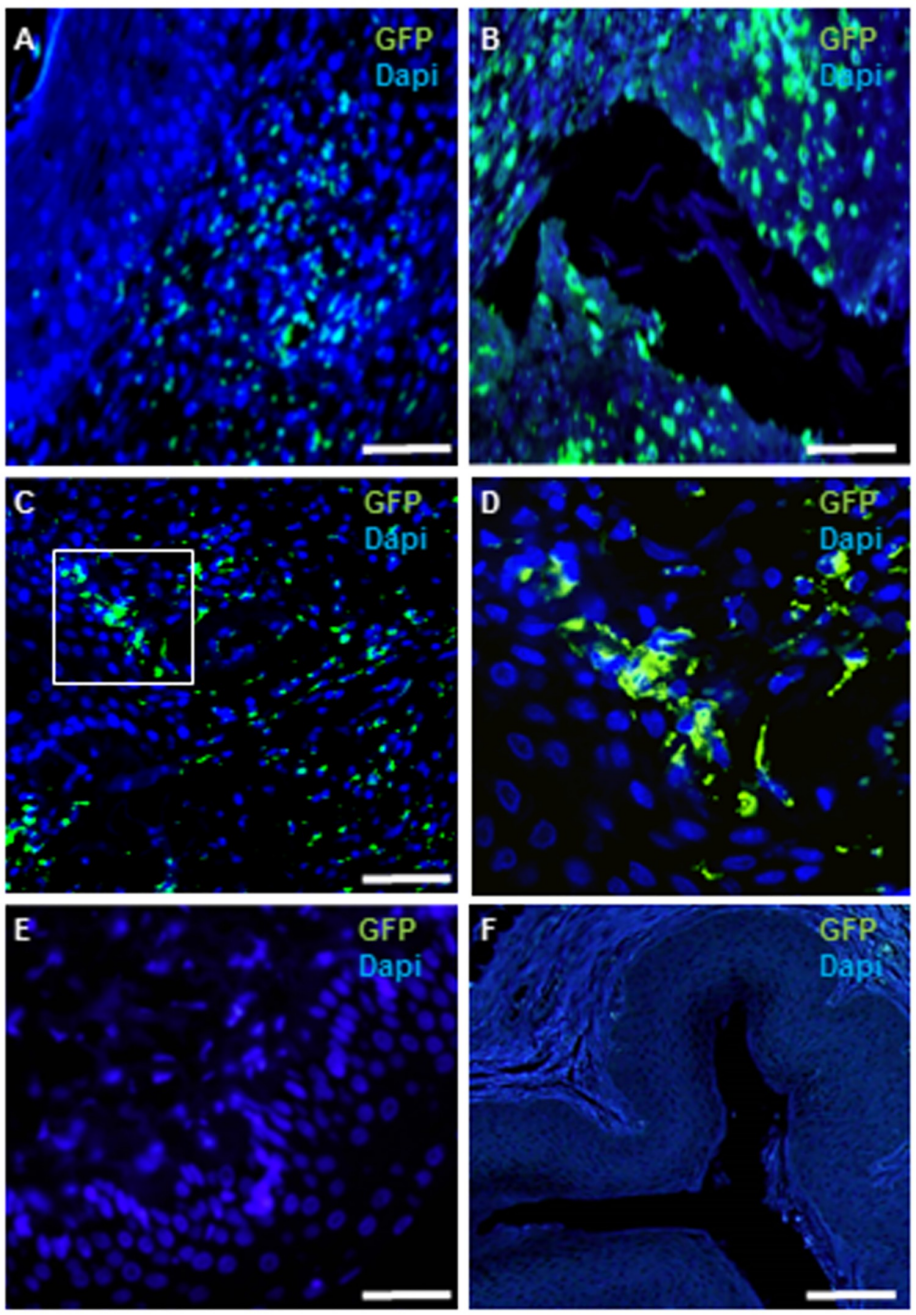
Survival of MSCs following systemic or local transplantation. On day three post injury, GFP- labeled cells homed to the injury site in the I.V. treated group (A). GFP positive cells were also evident at day three in the locally treated group (B). On day seven, GFP transplanted cells could be detected in the I.V. treated group (C). A higher magnification of the GFP labeled cells occupying the white square in C, is shown in D. In the locally treated rats, GFP transplanted cells could not be detected on day 7 (E). A group of rats (n = four rats) were transplanted systemically with MSCs, without making a vaginal incision. GFP-expressing cells were not detected in the vagina in this group (F). Scale bar A-B; D-E = 50 μm; C = 100 μm; F = 200 μm.

On day three post injury, GFP- labeled cells homed to the injury site in the I.V treated group (Fig 2A). GFP positive cells were also evident at day three in the locally treated group (Fig 2B). However, in the locally treated group, cells could not be found beyond this early time point (Fig 2E). In contrast to the local group, in the I.V. group, GFP transplanted cells were found in the vagina on day 7 (Fig 2C). A higher magnification image (marked by the white square in Fig 2C) of these transplanted GFP-positive cells is presented in Fig 2D. To confirm that the injury induced the migration of MSCs to the wound site, a group of rats (n = four rats) were transplanted systemically with MSCs, without making a vaginal incision. MSCs were not detected in the vagina in this group (Fig 2F). We could not detect MSCs in other organs (lung and liver) on day seven post transplantation (data not shown).

### Systemically transplanted MSCs differentiate into endothelial cells

**Fig 3:**
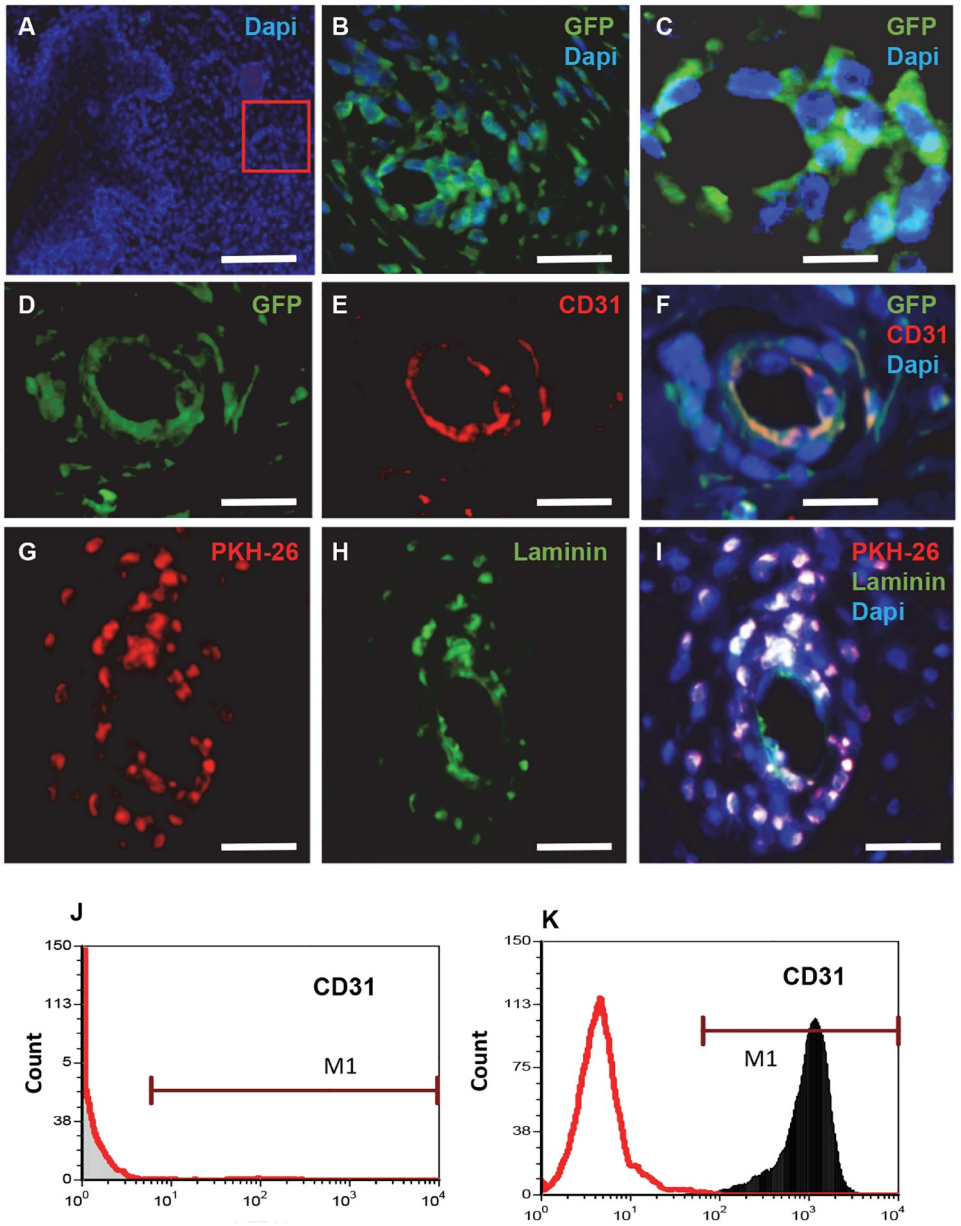
Systemically transplanted MSCs differentiate into endothelial cells. (A) DAPI stained section at seven days post injury and transplantation. Adjacent to the incision site a capillary-like structure is observed (red square). Fluorescence image of this capillary-like structure shows that it mostly made of GFP-expressing cells (B). A higher magnification of B is demonstrated in (C). Sections were stained with anti- GFP (D) and the endothelial marker CD31 (E). The co-localization of CD31 and GFP is shown in (F). PKH-26 transplanted cells (G), expressed laminin (H) and the co-localization of PKH-26 and laminin is shown in (I). FACS analysis prior to transplantation showed that the MSC population did not include CD31- expressing cells (J). Jurkat T cells decorated with anti-CD31, were used as positive controls (K). Scale bar: A = 500 μm; B, D, E, F = 100 μm; C, G, H, I = 50 μm.

Analysis on day 7 after systemic transplantation, showed GFP and PKH-26 labeled cells distributed within the lamina propria at the injury site. Interestingly, some of the transplanted labeled cells were organized in capillary-like structures (Fig 3A–3C). To characterize these capillary-like structures we stained the sections with anti-CD31, an endothelial marker. Transplanted GFP+ MSCs in the capillary-like structure wall co-expressed CD31 (Fig 3D–3F) suggesting that the transplanted cells formed, at least in part, the capillary-like structure. In addition, capillary-like structure composed of PKH-26 labeled cells, expressed laminin, a major component of the basement membrane that surrounds endothelial cells in blood vessels (Fig 3G–3I). To rule out the possibility that the population of transplanted cells already contained endothelial precursor cells, we examined the expression of CD31 in cultured MSCs by FACS analysis. Prior to transplantation, the MSC cultures did not include CD31+ cells (Fig 3J). We used Jurkat T cells as positive control to the expression of CD31 (Fig 3K). These results suggested that the transplanted cells acquired an endothelial phenotype *in vivo*.

### I.V. transplanted MSCs form blood vessel structures on day 30 after transplantation

**Fig 4:**
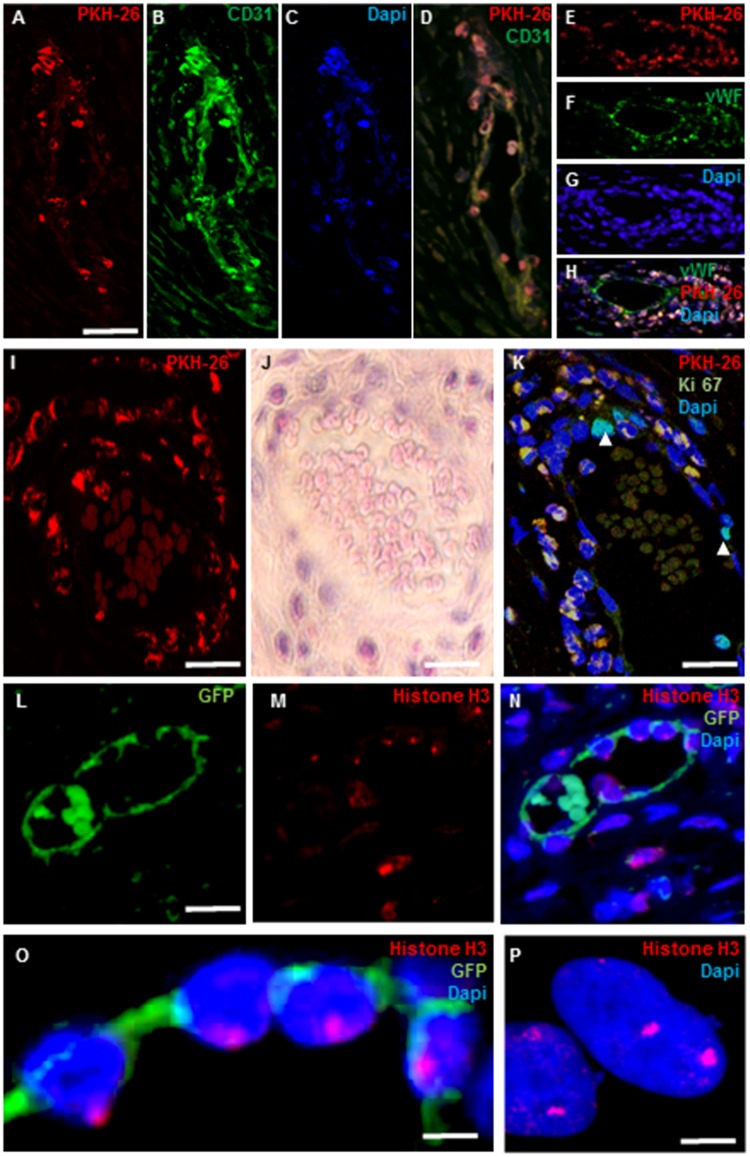
Systemically transplanted MSCs form blood vessel structures on day 30 after transplantation. Confocal microscopy showed that at thirty days after transplantation, PKH-26 transplanted cells (red) were organized in blood vessels-like structures (A) and were expressing CD31 (B). Nuclei are counterstained with DAPI (C). A merged image showing the co- localization of PKH-26 and CD31 is shown in (D). PKH-26 transplanted cells, organized in a capillary like structure (E), decorated with anti-vWF are demonstrated in (F). Nuclei are counterstained with DAPI (G). A merged image showing co-localization of PKH-26 and vWF (H). An example of a blood vessel composed of PKH-26 labeled cells is seen in (I). H&E staining of an adjacent section demonstrates the existence of enucleated erythrocytes in the vessel’s lumen (J). Proliferating cells expressing Ki67 (white arrows) were observed in the inner cell layer of blood vessel composed of PKH-26 transplanted cells. (K). Double staining with GFP (L) and anti trimethyl- Histone H3 (M) shows only one red focus in each cell. A merged picture is demonstrated in (N) and a higher magnification of N is shown in (O). Staining with anti trimethyl- Histone H3 in vitro of a hiPSC line with 93XXXXY karyotype that was formed by fusion between 46XX and 47XXY lines shows two red foci per cell (P). Scale bar: (A-D, F) 100 μm; (E) 500 μm; (G-L) 50 μm; (M-N) 5 μm.

Confocal imaging of the site of injury, 30 days after transplantation, confirmed that transplanted PKH-26+ cells (Fig 4A) within capillary-like structures co-expressed CD31 (Fig 4B; nuclei are counterstained with DAPI (Fig 4C)). The co-localization of PKH-26 and CD31 is shown in Fig 4D. Sections were also stained with anti von Willebrand factor antibody, an additional endothelial marker. PKH-26 positive cells (Fig 4E) express vWF (Fig 4F). Counterstaining with DAPI is demonstrated in Fig 4G. The co- localization of PKH-26 and vWF is demonstrated in Fig 4H. An example of a blood vessel composed of PKH-26 labeled cells is shown in Fig 4I. H&E staining of an adjacent section demonstrates the existence of enucleated erythrocytes in the vessel’s lumen, confirming its functionality (Fig 4J). Proliferation of cells expressing Ki67 was observed in the inner cell layer of the blood vessels that were composed of PKH-26 labeled cells (Fig 4K). Taken together, these results suggest that the transplanted cells acquired an endothelial phenotype *in vivo* and were capable to contribute to the formation of new blood vessels.

To rule out the occurrence of fusion between transplanted MSCs and the host endothelial cells, slides were stained with anti-trimethyl histone H3 (five rats per group, three slides from each rat were stained). This antibody reacts with the inactive X chromosome of females. Since the MSCs were derived from female rats, if fusion occurred, cells would be expected to have 2 inactivated x chromosomes [45,46], manifested by 2 red foci in each cell.

To confirm the validity of the system, we stained a karyotypically abnormal (93 XXXXY) induced human pluripotent stem cell (ihPSC) line. The line was formed as a result of fusion of two iPSC lines with 46XX and 47XXY karyotype. Staining with anti-trimethyl histone H3 revealed cells that clearly expressed two red foci (Fig 4P).

GFP transplanted cells with two red foci were not observed *in vivo* and all analyzed cells expressed one red focus. Transplanted GFP+ cells in the wall of a capillary-like structure (Fig 4L) expressing one red focus are demonstrated in Fig 4M. Co- localization of the Histone H3 red focuses within GFP positive cells is shown in Fig 4N. A higher magnification of these cells is shown in Fig 4O. Demonstration of one inactive X chromosome in the transplanted cells composing the wall of capillary-like structures supports the hypothesis that the transplanted MSCs differentiated into endothelial cells rather than fused with host endothelial cells.

### The effect of systemic MSCs transplantation on tissue vascularization

**Fig 5:**
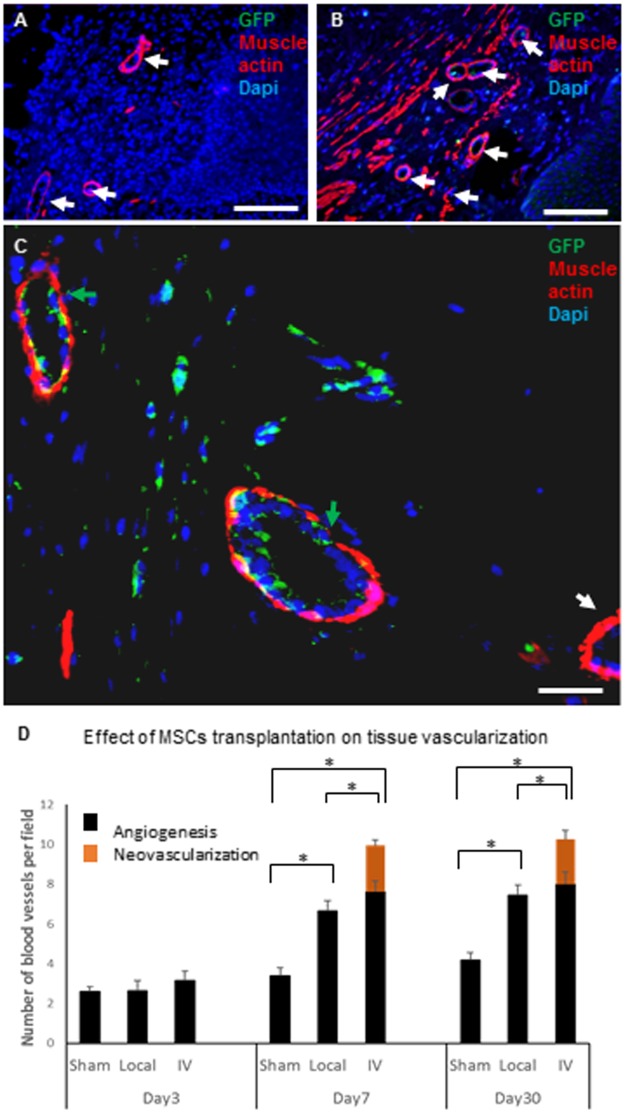
The effect of MSCs transplantation on tissue vascularization. To assess the effect of MSCs transplantation on tissue vascularization, vaginal sections from sham, local and I.V. transplanted rats were stained with anti-muscle actin and GFP antibodies. Representative images from day 7 of sham (A) and local (B) transplanted rats and of day 30 I.V. transplanted animals (C) are shown. The number of blood vessels without any staining to GFP (indicated with white arrows) per field at 10x magnification were analyzed (each group included five rats; three slides per rat; three fields were counted per slide). In addition, the number of blood vessels which had positive staining to GFP in the inner cell layer (green arrows) was analyzed. At days 7 and 30, MSCs transplantation induced angiogenesis (blood vessels without GFP) in both the local and systemically transplanted groups (D). Only in the systemically transplanted group, blood vessels composed of GFP-expressing cells, probably representing neovascularization involving the transplanted MSCs, were observed at 7 and 30 days post transplantation (C and D).*p<0.05. Scale bar: A-B = 500 μm; C = 50 μm.

To assess the effect of MSCs transplantation on tissue vascularization, slides were stained with anti-muscle actin and anti-GFP antibodies (Fig 5A–5C). The angiogenesis effect was evaluated by counting blood vessels which were positive to muscle actin but negative to GFP. The neovascularization effect was determined by counting blood vessels which were positive to muscle actin and composed of GFP positive cells (Fig 5C). Histogram representing this quantification is presented in Fig 5D. The angiogenic effect of MSCs transplantation was evident as early as 7 days and lasted until 30 days, the latest time point analyzed after transplantation (4.2± 0.37, 7.4± 0.52, 8± 0.66 blood vessels/field for sham, local and I.V. transplantation, respectively; P<0.05). In the systemically transplanted group, in addition to the trophic effect on angiogenesis, vascular structures composed of endothelial cells differentiated from GFP labeled transplanted MSCs were evident from day 7 (; 3.4 ± 0.42, 6.6± 0.5, 11.6± 0. 63 for sham, local and I.V. transplantation, respectively; P<0.05). These new vascular structures were evident only in the systemically transplanted group and constituted 23.7% ± 1.94 of the counted blood vessels on day 7 and 22% ±3.27 on day 30.

## Discussion

In this study, we have shown that systemic transplantation of bone-marrow derived MSCs after vaginal incision was associated with homing of the transplanted cells to the injury site and their survival for at least 30 days. MSCs transplantation induced the formation of new blood vessels by angiogenesis. In addition, the transplanted cells differentiated *in vivo* into endothelial cells within capillary-like structures.

The bone marrow consists of a heterogonous cell population, which includes hematopoietic stem cells, endothelial precursor cells (EPCs) and MSCs. The distinction between them relies on the expression of a different set of markers by each cell type [43]. The MSCs that were derived in this study expressed CD90 and CD29 and were negative for CD34, CD45 and CD11b/c. To rule out the existence of EPCs in our population of MSCs, the expression of CD31, which is a typical marker of EPCs but not of MSCs, was examined [33–35]. FACS analysis showed that the MSC populations that we used for transplantation did not include CD31 positive cells.

The vasculogenic properties of MSCs are of great interest in many pathological conditions, and whether MSCs are actually capable of differentiating *in vivo* into endothelial cells and promoting neovascularization, is still controversial [7,8,11,47]. It is well established that MSCs possess the ability to differentiate *in vitro* into endothelial cells [48,49] in the presence of an appropriate stimulating factors such as vascular endothelial growth factor (VEGF) and insulin-like growth factor 1 (IGF-1) [11,19,50]. MSCs that were transplanted after induction of endothelial differentiation *in vitro* formed neo-vascular structures *in vivo*, in SCID mice [51]. Studies of the potential of MSCs to treat cardiovascular diseases, have shown the ability of MSCs, that were not induced towards endothelial differentiation prior to engraftment, to form new blood vessels by differentiating into endothelial cells *in vivo* [52,53]. In contrast to these observations, other studies have shown poor viability and survival of the transplanted cells in the host tissue [54–56]. Our findings suggest that bone marrow derived MSCs that are transplanted systemically, without pre-treatment with endothelial inducing factors, migrate to vaginal injury site, survive and differentiate *in situ* into endothelial cells.

The new capillary-like structures that included endothelial cells originating from MSCs differentiation constituted 22% of all blood vessels observed in the vagina. This rate remained stable between days 7 and 30 post-injury. This is in accordance with previous studies [57–59] that demonstrated a role for stem cell-induced vascularization in the recovery processes. These studies showed a similar level of newly- formed blood vessel density, which was associated with a beneficial effect on the wound healing course following injury.

The endothelial phenotype of the transplanted cells *in vivo*, could potentially result from fusion between transplanted MSCs and host endothelial cells [60,61]. In order to exclude this possibility, we stained our specimens with anti-trimethyl histone H3, which can demonstrate the presence and number of inactive X chromosomes in cells. In our study, MSCs with female genotype were transplanted into female rats and anti-trimethyl histone H3 staining showed only one inactive X chromosome in GFP positive endothelial cells. This implies that the GFP+ endothelial cells resulted from direct differentiation of the transplanted MSCs rather than from cell fusion.

Potential therapeutic effect of MSCs transplantation in PFD was suggested on the basis of two mechanisms; paracrine and anti- inflammatory effect [35,62] and differentiation of the transplanted cells into the host tissue [63,64]. Pre-clinical studies using different models of SUI have shown results supporting both of these mechanisms. These studies were translated into clinical trials [65–67]. However, only limited number of studies examined the effect of MSCs transplantation on the vaginal connective tissue. MSCs from different sources, including the bone marrow [30,32,38,68], adipose tissue [69], muscle [70] and endometrium [9,33,71] were transplanted into the vagina. Interestingly, in these studies, cells did not survive in the vaginal tissue for long duration, suggesting that any beneficial effect that was perceived, was mainly due to their trophic effect.

In line with these studies, we observed poor survival of the MSCs following local transplantation to a vaginal injury site. Nevertheless, our study is the first to demonstrate vaginal engraftment and long- term survival of MSCs for at least 30 days, after systemic transplantation. MSCs were not found in other organs (lung and liver) at later time points following transplantation. Moreover, to our knowledge, this is the first study to show the differentiation of MSCs into endothelial cells *in vivo* in the vagina.

Aging is one of the main contributing factors for PFD. Many of the patients suffering from PFD are middle aged or elderly. Studies have shown that aging has a detrimental effect on wound healing processes, partly due to reduced vascularity [72,73]. The suboptimal results of surgery for PFD may be attributable to aging related delayed wound healing. Additional studies are needed to determine whether the increased vascularity caused by MSCs transplantation improve the quality of vaginal healing following surgery in elderly individuals.

These findings pave the way to further studies of the potential role of MSCs in improving surgical outcome in women with pelvic floor disorders.

## Supporting information

S1 Fig Characterization of MSCs *in vitro*. (A) Cells were isolated from the bone marrow and plated in two petri dishes. (B) Few spindle like cells are starting to appear 48 hours after plating, marked with the black arrows. (C) A higher magnification of one attached cell 48 hours after plating. (D) Three weeks after plating, cells had a fibroblast-like appearance and reached 85–90% confluence. (E) Cells were grown with MSC go Osteogenic XF^™^ for 21 days and then stained with Alizarin red S. The staining of mineralized bone matrix is shown in red. (F) Cells were grown with MSC go Chondrogenic XF^™^ for 21 days. Cartilage containing aggrecans stained blue after incubation with alcian blue. (G) MSC were grown with MSC go Adipogenic XF^™^ for 21 days and stained with Oil red-O. Intracellular lipid droplets are seen in E and not in (H), where cells were grown in the control medium. (I) Representative figures from FACS analysis for CD90, CD29, CD34, CD45 and CD11b/c are shown. The cells stained positive for CD90 and CD29 and negative for the typical hematopoietic markers CD34, CD45 and CD11b/c. Scale bar: A, B, D, E, G, and H = 200μm; C, F = 100 μm.
(PPT)
