# Supplementary figures and images for: Systemically transplanted mesenchymal stem cells induce vascular-like structure formation in a rat model of vaginal injury
Source: PLoS One. 2019 Jun 13;14(6):e0218081. doi: 10.1371/journal.pone.0218081 (PMC6563972; doi:10.1371/journal.pone.0218081)

## Slide 1
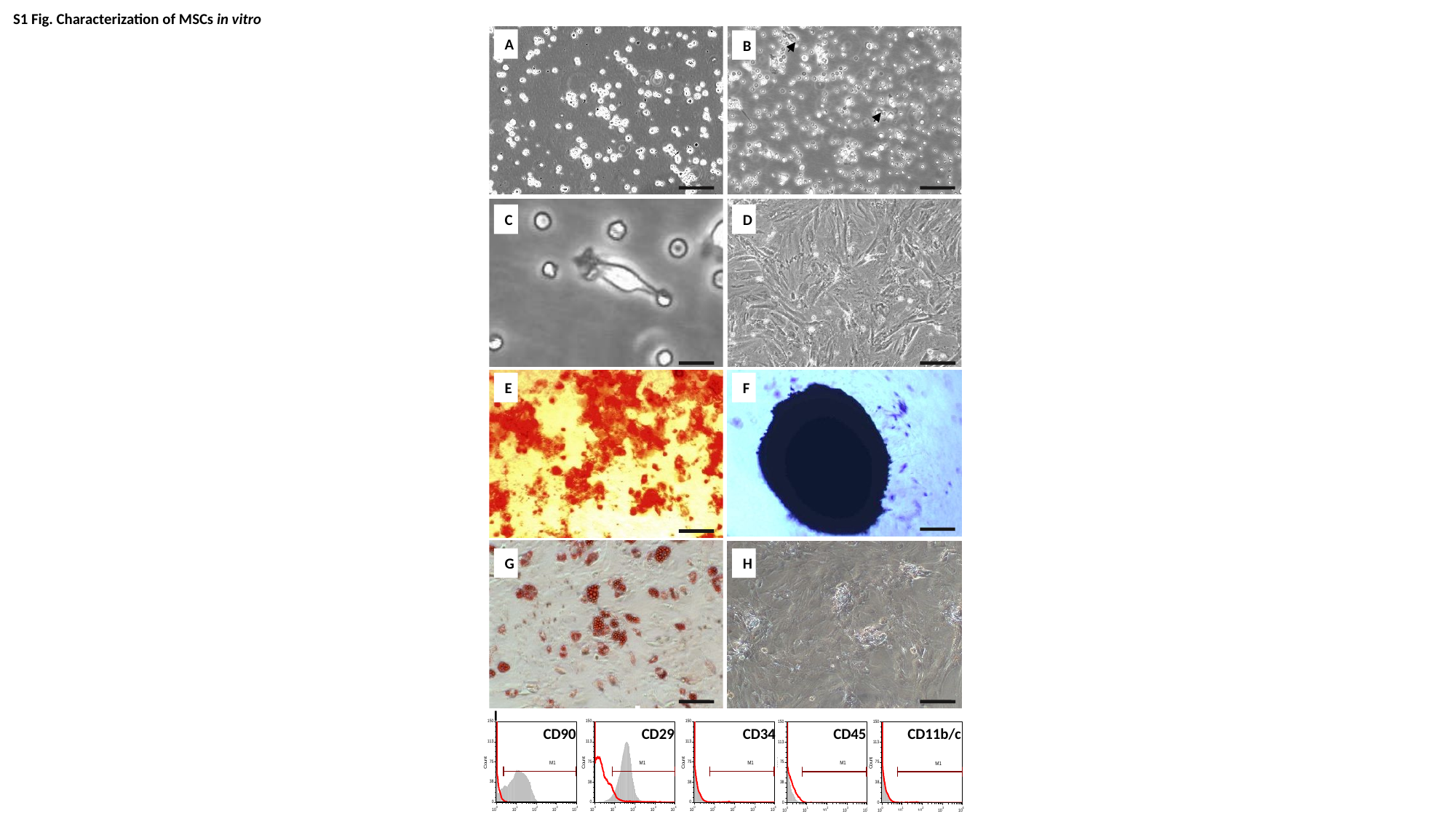

S1 Fig. Characterization of MSCs in vitro
A
B
C
D
E
F
G
H
I
CD90
CD29
CD34
CD45
CD11b/c

Supplement: S1 Fig — (A) Cells were isolated from the bone marrow and plated in two petri dishes. (B) Few spindle like cells are starting to appear 48 hours after plating, marked with the black arrows. (C) A higher magnification of one attached cell 48 hours after plating. (D) Three weeks after plating, cells had a fibroblast-like appearance and reached 85–90% confluence. (E) Cells were grown with MSC go Osteogenic XF™ for 21 days and then stained with Alizarin red S. The staining of mineralized bone matrix is shown in red. (F) Cells were grown with MSC go Chondrogenic XF™ for 21 days. Cartilage containing aggrecans stained blue after incubation with alcian blue. (G) MSC were grown with MSC go Adipogenic XF™ for 21 days and stained with Oil red-O. Intracellular lipid droplets are seen in E and not in (H), where cells were grown in the control medium. (I) Representative figures from FACS analysis for CD90, CD29, CD34, CD45 and CD11b/c are shown. The cells stained positive for CD90 and CD29 and negative for the typical hematopoietic markers CD34, CD45 and CD11b/c. Scale bar: A, B, D, E, G, and H = 200μm; C, F = 100 μm. (PPT) [file pone.0218081.s001.ppt]
